# Combined assisted bone age assessment and adult height prediction methods in Chinese girls with early puberty: analysis of three artificial intelligence systems

**DOI:** 10.1007/s00247-022-05569-3

**Published:** 2022-12-28

**Authors:** Shurong Huang, Zhe Su, Shuangyi Liu, Jinfeng Chen, Qiru Su, Huiping Su, Yue Shang, Yanhua Jiao

**Affiliations:** 1 Department of Endocrinology, Shenzhen Children’s Hospital, No. 7019, Yitian Road, Futian District, Shenzhen, 518038 Guangdong Province People’s Republic of China; 2 School of Medicine, Southern University of Science and Technology, Shenzhen, Guangdong China; 3 Department of Clinical Research, Shenzhen Children’s Hospital, Shenzhen, Guangdong China

**Keywords:** Adult height, Artificial intelligence, Bayley–Pinneau, Bone age, Child, Early puberty, Greulich-Pyle, Radiography, Tanner–Whitehouse

## Abstract

**Background:**

The applicability and accuracy of artificial intelligence (AI)-assisted bone age assessment and adult height prediction methods in girls with early puberty are unknown.

**Objective:**

To analyze the performance of AI-assisted bone age assessment methods by comparing the corresponding methods for predicted adult height with actual adult height.

**Materials and methods:**

This retrospective review included 726 girls with early puberty, 87 of whom had reached adult height at last follow-up. Bone age was evaluated using the Greulich–Pyle (GP), Tanner–Whitehouse (TW3–RUS) and China 05 RUS–CHN (RUS-CHN) methods. Predicted adult height was calculated using the China 05 (CH05), TW3 and Bayley–Pinneau (BP) methods.

**Results:**

We analyzed 1,663 left-hand radiographs, including 155 from girls who had reached adult height. In the 6–8- and 9–11-years age groups, bone age differences were smaller than those in the 12–14-years group; however, the differences between predicted adult height and actual adult height were larger than those in the 12–14-years group. TW3 overestimated adult height by 0.4±2.8 cm, while CH05 and BP significantly underestimated adult height by 2.9±3.6 cm and 1.3±3.8 cm, respectively. TW3 yielded the highest proportion of predicted adult height within ±5 cm of actual adult height (92.9%), with the highest correlation between predicted and actual adult heights.

**Conclusion:**

The differences in measured bone ages increased with increasing bone age. However, the corresponding method for predicting adult height was more accurate when the bone age was older. TW3 might be more suitable than CH05 and BP for predicting adult height in girls with early puberty. Methods for predicting adult height should be optimized for populations of the same ethnicity and disease.

## Introduction

Early puberty is the normal bias between precocious puberty and normal puberty; the age at puberty is between 0 and ± 2 standard deviations of normal age within the same ethnic group, or 3rd~50th percentiles [1]. According to a national survey in China, early puberty is defined as the onset of puberty in girls between 7.1 years and 9.2 years of age [1, 2]. Worldwide, the population with early puberty is increasing as the age of puberty onset in children decreases [2–4]. Girls are >10 times more likely to visit a doctor than boys because girls’ sex characteristics are easier to identify than boys’, inducing parental anxiety [5–7]. Most girls with early puberty do not require drug intervention [7]. A small proportion of girls with impaired predicted adult height or rapid progression require therapy with gonadotropin-releasing hormone analog. The accuracy of bone age assessment and prediction of adult height are important bases for informed treatment [8].

The Tanner–Whitehouse (TW3–RUS) [9] and Greulich–Pyle (GP) methods [10] are internationally accepted methods for evaluating bone age [11]. The TW3–RUS and GP methods were developed in the last century and are based on Western Caucasian children. To eliminate racial disparities, the China 05 (CH05) method has been proposed for Chinese children [12, 13]. The CH05 method is a general term for three methods, including TW3–Chinese RUS, TW3–Chinese carpal and RUS–CHN. The authors of CH05 believe that the RUS–CHN method is more applicable in the medical field than the other two methods [12]. The TW3–RUS, GP and CH05 RUS–CHN methods have their corresponding systems for predicting adult height, namely the TW3, Bayley–Pinneau (BP) and CH05 methods, respectively [9, 12, 14]. The accuracy of the methods for predicting adult height can be affected by race, secular changes and disease state, which have an impact on skeletal development and adult height [15, 16]. Even when the TW3 and BP methods are used in Caucasian children, their accuracies in children with delayed puberty and growth hormone deficiency require further validation [17–20]. Furthermore, girls with early puberty have a distinct pubertal growth pattern [21]. There is a lack of studies comparing and analyzing the accuracy of different bone age assessment methods and adult height prediction methods in girls with early puberty in China.

Traditional manual evaluation of bone age is not only subjective but also time-consuming and labor-intensive. Large-scale studies comparing bone age assessment and adult height prediction methods are lacking. With the development of science and technology, the use of artificial intelligence (AI) to evaluate bone age in the medical field has become increasingly common [22–24]. AI systems may achieve results comparable to those of traditional manual bone age evaluation and have the advantages of being objective, highly efficient and labor-saving [23, 25–28]. In this study, based on the clinical data of a large sample of girls with early puberty, we used AI systems to replace the manual evaluation of bone age and compared the TW3–RUS, GP and RUS–CHN methods to evaluate the bone age of girls with early puberty. Using the adult height of girls as a reference, we compared the accuracy of the TW3, BP and CH05 methods for predicting adult height in girls with early puberty. Predicted adult height is calculated based on the bone age and height at the time of radiographic examination. Therefore, accuracy of the adult height prediction reflects the accuracy of the bone age assessment method. In short, we analyzed the performance of three bone age assessment methods with AI assistance by comparing the predicted adult height with the actual adult height. The results of this study may provide a reference for pediatricians in making clinical decisions and offer a research basis for further improvement or development of bone age assessment and adult height prediction methods.

## Materials and methods

### Subjects

In this retrospective study, we recruited girls who were outpatients at the Endocrinology Department at Shenzhen Children’s Hospital between June 2015 and December 2021 who visited because of premature breast development. All girls included in the study met only one criterion: breast development (Tanner stage ≥B2) between the ages of 7.1 years and 9.2 years. Girls who were excluded from the study met at least one of the following criteria: (a) they had mammary gland hyperplasia, osteochondral dysplasia, genetic metabolic diseases, other endocrine diseases (including growth hormone deficiency and thyroid dysfunction) or extreme obesity (body mass index ≥120% of the 95th percentile); (b) they were born small for gestational age or premature; or (c) they had undergone treatment with traditional Chinese medicine, gonadotropin-releasing hormone analog or growth hormone.

**Fig. 1:**
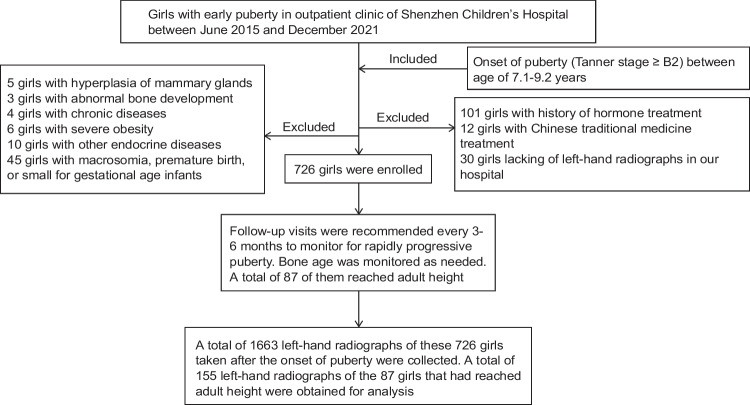
Research design flow chart

Follow-up visits were recommended every 3–6 months to monitor for rapidly progressive puberty. Bone age was monitored as required. All left-hand radiographs obtained after the onset of puberty (Tanner stage ≥B2) were collected for the study. Information such as adult height and other data were obtained through clinical or telephone follow-up (Fig. 1). Adult height was defined as the point when height velocity was <2.0 cm/year during the last year and >15 years of age.

Parents or guardians of the girls gave written informed consent or verbal consent to participate in the study. All the methods involved in the study complied with the relevant guidelines or standards. This study was approved by the ethics committee of our hospital (N.2021036).

### Physical examination

We obtained physical examination information from hospital medical records. Endocrinology Department pediatricians with more than 3 years of work experience evaluated secondary sexual characteristics using Tanner staging. The standing height (centimeters) was measured using a Seca 274 (Hamburg, Germany) height meter (sensitivity, 0.1 cm) by pediatrician assistants with more than 1 year of work experience. Every girl was measured twice, and the mean value calculated. The girls were bare-footed and wore shorts and t-shirts. Follow-up adult height was reported by the girls’ parents, who were taught on the proper method of measuring height.

### Bone age assessment and adult height prediction

Left-hand radiographs were obtained by trained radiologists at our hospital. For each left-hand radiograph, we used an AI system to evaluate bone age according to the GP [10], TW3–RUS [9] and RUS–CHN methods [12]. These automated AI systems are called intelligent diagnosis systems for child growth and development (Deepwise Artificial Intelligence Lab; Beijing, China) [27]. For the 87 girls who reached adult height, we calculated predicted adult height and compared it with actual adult height. Bone age determined at the time the girls’ radiographs were taken was highly correlated with the proportion of girls achieving adult height. Therefore, after determining the bone age and height of the participants, we could calculate the predicted adult height using the formulas according to TW3, CH05 and BP methods [9, 12, 14].

The TW3–RUS method is a scoring method to assess the maturity scores of the radius, ulna and 11 metacarpals and phalanges. The total score is then calculated and converted to bone age. Based on the TW3–RUS method, the RUS–CHN method adds 2–6 maturity indicators for each bone (47 in total), while the weight distribution of the bones remains unchanged. For example, the developmental grade of the radius is divided into 9 grades (A–I) in the TW3 method but is increased to 15 grades (A–O) in the RUS–CHN method. In both methods, fully mature bone is assigned a score of 1,000 points, but the corresponding bone ages are different. In the RUS–CHN method, the corresponding bone age is 17 years, and in the TW3–RUS method, it is 15 years. The method for predicting adult height corresponding to the RUS–CHN method was not based on the TW3 method, but on the BP method [12]. Predicted adult height by the CH05 method was calculated according to the corresponding adult height percentage.

### Statistical analysis

Results are expressed as the mean ± standard deviation (SD). We used the coefficient of variance (CoV) to describe the degree of dispersion of predicted adult height when using the three different methods. The calculation formula was CoV=(SD/mean) × 100%. We used the Kolmogorov–Smirnov test to determine the compliance of the variables to a normal distribution. Bone ages and predicted adult heights measured by the three methods were compared using the Friedman test and Wilcoxon rank sum test, respectively. We used Spearman rank correlation to estimate the linear association between adult height and predicted adult height using the three prediction methods. Two-tailed *P*<0.05 was considered statistically significant. All statistical analyses were conducted using the SPSS statistical software (version 23.0; IBM, Armonk, NY).

## Results

### Patients and left-hand radiographs

A total of 726 girls were included in the study, and 1,663 left-hand radiographs were available for analysis. The age range at the time of radiography was 7.1–14.8 years. Among the girls, 87 (11%) reached adult height at follow-up, and they had 155 left-hand radiographs available for analysis.

### Comparison among artificial-intelligence-assisted bone age assessment methods

**Fig. 2:**
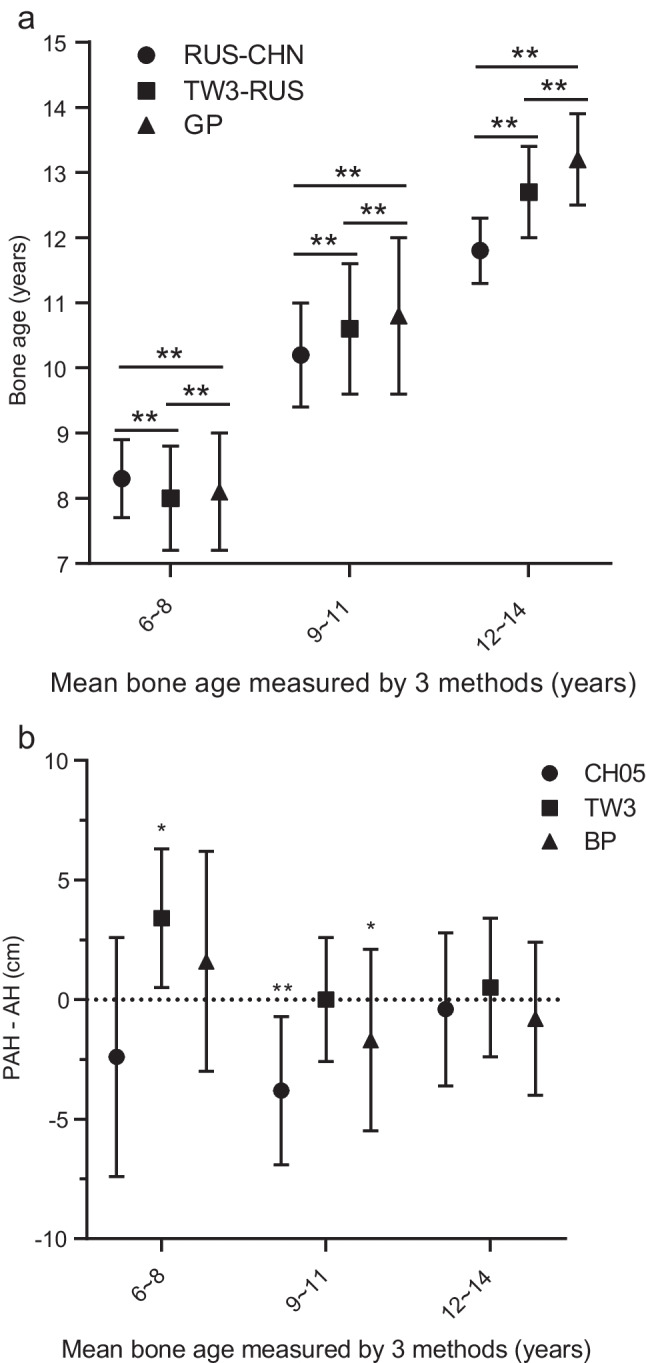
Comparison of methods for assessing bone age and predicting adult height. **a** Mean error plot of bone age (BA) obtained by the three BA evaluation methods: RUS–CHN, Tanner–Whitehouse (TW3–RUS) and Greulich–Pyle (GP). The abscissa is the average grouping of BA obtained by the three methods. The differences in BAs were statistically significant in all groups (*P*<0.001). **P<0.001. **b** The mean differences between the predicted adult height (PAH) and adult height (AH) obtained by the three adult height prediction methods — China 05 (CH05), Tanner–Whitehouse (TW3) and Bayley–Pinneau (BP) — among the BA groups. * Statistically significant at *P*<0.05. ** Statistically significant at *P*<0.001

Because there is no gold standard for bone age assessment, we divided the radiographs into three groups based on the mean bone age obtained by the three bone age assessment methods: a 6–8-years bone age group (*n*=424), a 9–11-years bone age group (*n*=1,121) and a 12–14-years bone age group (*n*=118) (Table 1; Fig. 2). In the 6–8-years bone age group, the bone age measured by the TW3–RUS method was the smallest (*P*<0.05), while the bone age measured by the RUS–CHN method was the largest (*P*<0.05). However, with bone age >9 years, the bone age measured using the RUS–CHN method was the smallest (*P*<0.05), while the bone age measured using the GP method was the largest (*P*<0.05). The difference among the bone ages measured by the three methods increased with increasing bone age (Table 1; Fig. 2). The difference in bone age obtained by the RUS–CHN and GP methods and the difference in bone age obtained by the RUS–CHN and TW3–RUS methods were >1 year when the bone age was 11.3 years and 12.5 years, respectively.

**Table 1:** Comparison of mean bone age (BA) obtained by the three BA evaluation methods

	*n*	RUS–CHN (years)	TW3–RUS (years)	GP (years)
6–8 years BA	424	8.3±0.6^a,b^	8.0±0.8^b^	8.1±0.9
9–11 years BA	1,121	10.2±0.8^a,b^	10.6±1.0^b^	10.8±1.2
12–14 years BA	118	11.8±0.5^a,b^	12.7±0.7^b^	13.2±0.7

*GP* BA measured by Greulich–Pyle, *RUS–CHN* BA measured by RUS*–*CHN, *TW3–RUS* BA measured by Tanner–Whitehouse

^a^Statistically different from BA measured by Tanner–Whitehouse (*P*<0.001)

^b^Statistically different from BA measured by Greulich–Pyle (*P*<0.001)

### Comparison between adult height prediction methods

Radiographs of girls who had reached adult height were divided into the 6–8-years bone age group (*n*=12), 9–11-years bone age group (*n*=108) and 12–14-years bone age group (*n*=35). In the younger bone age groups (6–8 years and 9–11 years), the differences in the bone age evaluated by the three methods were smaller than those in the older bone age group (12–14 years); however, the differences in adult height predictions using the corresponding methods (i.e. TW3, BP and CH05) and the actual adult heights (predicted adult height minus adult height) were larger among methods in younger than in the oldest bone age group (12–14 years) (Table 2; Fig. 2). This indicates that the differences in bone age assessed by the three bone age evaluation methods were not equivalent to the differences in predicted adult height obtained by their corresponding adult height prediction methods.

**Table 2:** Differences between the adult height (AH) and predicted adult height (PAH) obtained by the three adult height prediction methods

	*n*	PAH_CH05_ minus AH (cm)	PAH_TW3_ minus AH (cm)	PAH_BP_ minus AH (cm)
Total	155	−2.9±3.6^b^	0.4±2.8	−1.3±3.8^b^
Grouped by bone age (BA)				
6–8 years BA	12	−2.4±5.0	3.4±2.9^a^	1.6±4.6
9–11 years BA	108	−3.8±3.1^b^	0.0±2.6	−1.7±3.8^a^
12–14 years BA	35	−0.4±3.2	0.5±2.9	−0.8±3.2

^a^Difference between PAH and AH was statistically significant (*P*<0.05)

^b^Difference between PAH and AH was statistically significant (*P*<0.001)

*PAH*_*BP*_ predicted adult height calculated by Bayley–Pinneau, *PAH*_*CH05*_ predicted adult height calculated by China 05, *PAH*_*TW3*_ predicted adult height calculated by Tanner–Whitehouse

**Fig. 3:**
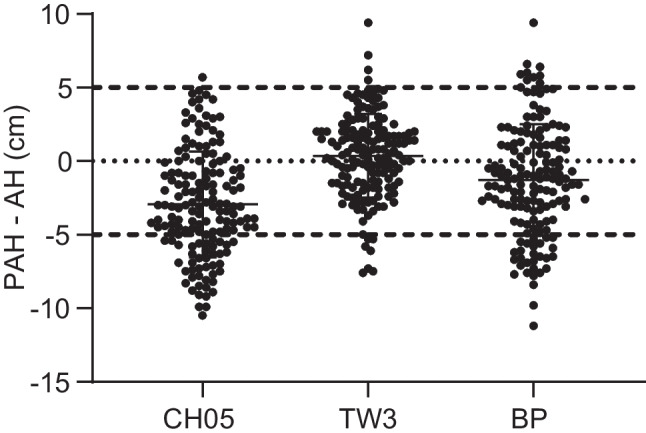
Scatter plot shows the differences between predicted adult height (PAH) and adult height (AH) obtained by the China 05 (CH05), Tanner–Whitehouse (TW3) and Bayley–Pinneau (BP) methods. The long and short horizontal lines represent the mean and standard deviation of the differences in PAH and AH, respectively

**Fig. 4:**
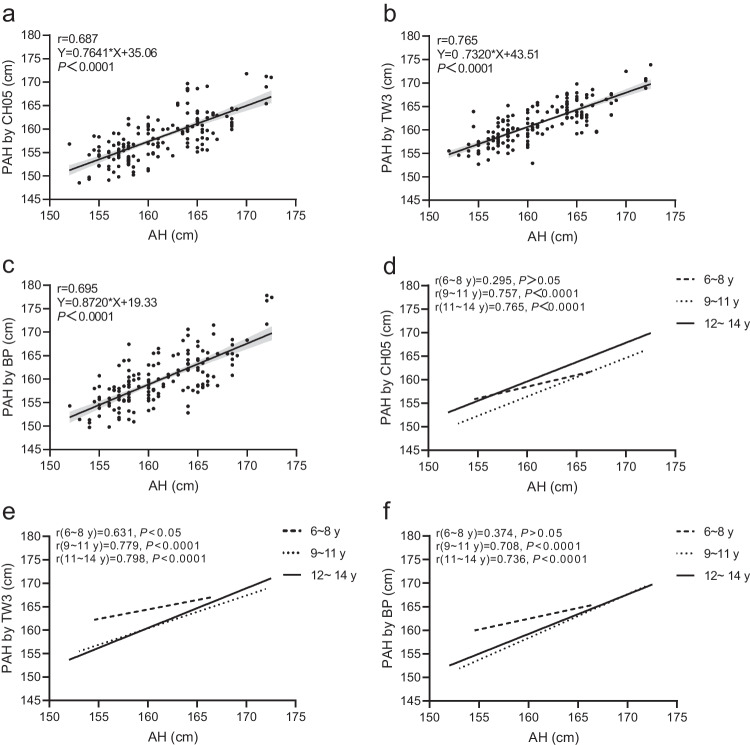
Correlation between adult height (AH) and predicted adult height (PAH). **a–c** AH and PAH as compared among the China 05 (CH05) (**a**), Tanner–Whitehouse (TW3) (**b**) and Bayley–Pinneau (BP) (**c**) methods. **d–f** Correlation of overall AH and PAH obtained by the three methods according to three bone age groups: CH05 (**d**), TW3 (**e**) and BP (**f**). Bone age here is the average bone age obtained by the RUS–CHN, TW3–RUS, and Greulich–Pyle (GP) methods. *P*<0.05 is significant

The average difference between predicted adult height and adult height for the TW3 method (0.4±2.8 cm) was not significantly different (*P*>0.05), whereas the average differences between predicted adult height and adult height for the CH05 (−2.9±3.6 cm) and BP (−1.3±3.8 cm) methods were significantly different (*P*<0.001). The CoV is useful for comparing the degree of variation between two sets of data, excluding the effect of possible differences between the means. The CoV of predicted adult height obtained by the TW3 method was the smallest (2.6%), followed by the CH05 (3.1%) and BP (3.4%) methods. The highest proportion of predicted adult height within ±5 cm of adult height was obtained using the TW3 method (92.9%), followed by the BP (76.0%) and CH05 (72.3%) methods. As demonstrated by the scatter plot (Fig. 3), the TW3 method has higher accuracy and precision in predicting the adult height of girls with early puberty than the other two methods. The correlation between predicted adult height and adult height obtained by the TW3 method was the highest (r=0.77, *P*<0.0001), followed by the BP (r=0.70, *P*<0.0001) and CH05 (r=0.69, *P*<0.0001) methods (Fig. 4).

The Spearman rank correlation coefficients of predicted adult height and adult height obtained by the three methods in the 6–8-years bone age group were smaller than those in the 9–11- and 12–14-years bone age groups (Fig. 4), indicating that the accuracy of predicting adult height in girls with early puberty and younger bone age is poorer than that in older girls with early puberty.

### Analysis of the failure cases of adult height prediction

To gain more insight into possible limitations of AI-assisted bone age assessment and adult height predictions, we selected nine cases with the largest differences between the predicted adult height and actual adult height (Table 3). Cases 1–3, 4–6 and 7–9 had the largest differences between the adult height and predicted adult height as predicted by the CH05, TW3 and BP methods, respectively. The factors that influence the accuracy of adult height prediction are complex. From these nine cases, we can conclude that possible factors included predicting height at a younger bone age (cases 1, 6 and 7), advancing bone age (bone age is >1 year older than age, cases 2, 3, 5, 8 and 9) and individual differences (cases 1, 2, 4 and 5).

**Table 3:** Characteristic of the nine failure cases of adult height prediction^a^

	Case 1	Case 2	Case 3	Case 4	Case 5	Case 6	Case 7	Case 8	Case 9
Age (y)	7.9	9.1	9.3	12.2	12.2	7.5	9.2	8.4	9.0
BA_RUS–CHN_ (y)	8.8	11.2	10.8	11.6	12.0	7.9	8.5	9.9	10.6
BA_TW3–RUS_ (y)	8.4	11.3	11.2	12.1	13.8	7.3	8.1	9.1	10.6
BA_GP_ (y)	8.8	12.0	11.0	12.0	13.5	7.9	7.8	11.0	12.0
BA_RUS–CHN_ – age (y)	0.9	2.1	1.5	−0.6	−0.2	0.4	−0.7	1.5	1.6
BA_TW3–RUS_ – age (y)	0.5	2.2	1.9	−0.1	1.6	−0.2	−1.1	0.7	1.6
BA_GP_ – age (y)	0.9	2.9	1.7	−0.2	1.3	0.4	−1.4	2.6	3.0
AH (cm)	165.0	165.5	166.0	167.0	160.5	154.5	158.0	164.0	164.0
Target height (cm)	155.5	159.0	160.0	159.0	153.0	155.0	158.5	161.5	161.5
PAH_CH05_ – AH (cm)	−9.9	−9.9	−10.5	−4.1	−5.8	1.0	4.2	−7.8	−7.0
PAH_TW3_ – AH (cm)	−1.2	−2.9	−5.0	−7.5	−7.6	9.4	7.2	2.0	−1.5
PAH_BP_ – AH (cm)	−7.8	−7.3	−6.5	−1.6	−6.8	3.5	9.4	−11.2	−9.8

*AH* adult height, *BA* bone age, *BA*_*GP*_ bone age measured by Greulich–Pyle, *BA*_*RUS–CHN*_ bone age measured by RUS–CHN, *BA*_*TW3*–*RUS*_ bone age measured by Tanner–Whitehouse (TW3–RUS), *PAH*_*BP*_ predicted adult height calculated by Bayley–Pinneau, *PAH*_*CH05*_ predicted adult height calculated by China 05, *PAH*_*TW3*_ predicted adult height calculated by Tanner–Whitehouse, *y* years

^a^Cases 1–3, 4–6 and 7–9 had the largest difference between the AH and PAH as predicted by China 05 (CH05), Tanner–Whitehouse (TW3) and Bayley–Pinneau (BP), respectively. Possible factors that influenced the accuracy of AH prediction included predicting height at a younger BA (cases 1, 6 and 7), advancing BA (BA is >1 year older than age, cases 2, 3, 5, 8 and 9) and individual differences (cases 1, 2, 4 and 5). Target height is mid-parental height minus 6.5 cm

## Discussion

The regularity and accuracy of different bone age assessment and adult height prediction methods have not been verified in various ethnic and disease groups. This study is the first to describe and compare AI-assisted RUS–CHN, TW3–RUS and GP methods in a population of girls with early puberty in China. Furthermore, we compared the accuracy of the corresponding adult height prediction methods (i.e. CH05, TW3 and BP) using actual adult height to evaluate the clinical applicability of the three bone age assessment systems. Our study revealed that the differences among the bone ages as measured by the three methods increased with increasing bone age, but the corresponding methods for predicting adult height were more accurate when bone age was older. Compared with the CH05 and BP methods, the TW3 method was more suitable for predicting adult height in girls with early puberty. The research results can provide a reference for pediatricians in making diagnostic and treatment decisions and offer a research basis for improvement or development of methods.

The GP method is an atlas containing reference images. Bone age assessment is performed by comparing the participants’ (left)-hand radiographs with the reference images in the atlas. The difference in bone age between two adjacent reference maps was 0.25–1.08 years. Therefore, although the GP method takes less time to evaluate bone age, it is not sufficiently accurate, especially with a bone age of >6 years. The TW3–RUS and RUS–CHN methods assess the maturity scores of 13 bones. The total score is then calculated and converted to bone age. Therefore, the TW3–RUS and RUS–CHN methods are more precise than the GP method, but they are more time-consuming. This is exacerbated by the increased clinical need for bone age assessment as the population with early puberty increases. AI-assisted bone age assessment can mitigate time costs. However, the accuracy of the three AI-assisted bone age assessment methods has not been verified in girls with early puberty. At present, there is no gold standard for evaluating the accuracy of bone age; therefore, we used the adult height of girls as the reference standard to evaluate which adult height prediction method corresponded best with bone age evaluation in girls with early puberty.

Differences among the bone age assessment methods have been demonstrated in many countries and ethnic groups; however, previous studies have not explained the significance of these differences through the assessments’ corresponding methods for predicting adult height. A study of 2,053 girls in China showed that in healthy girls ages 7–16 years, the bone age measured by the GP method was greater than that measured by the RUS–CHN method, and the difference increased with age [29]. In a study using the BoneXpert automatic software, the bone age measured using the GP method was greater than that measured using the TW3 method [28]. In this study, the bone age measured by the GP method was larger than that measured by the RUS–CHN method with a bone age of >9 years, and was larger than that measured by the TW3–RUS method with a bone age of 6–14 years. Zhang et al. [30] evaluated racial differences in the skeletal growth patterns of Asian, Caucasian and Hispanic children in the United States. Using the GP method to evaluate the bone age, they reported that the bone age of Asian girls was on average 0.7 years older than that of Caucasian girls, and the difference between the two was greatest at 10–15 years of age [30]. This might reflect the fact that the difference in the rate of skeletal maturation between Asian and Caucasian girls increases with the progression of puberty. However, Zhang et al. [30] did not further compare whether differences in bone age assessment methods affect the accuracy of the corresponding adult height prediction methods. In our study, we demonstrated that although the difference between the bone ages measured by the three methods increased with increasing bone age, the corresponding adult height prediction method was more accurate with older bone age. Therefore, we do not recommend changing the bone age assessment methods during long-term monitoring of bone age in children. When making diagnosis and treatment decisions, it is necessary to consider bone age, predicted adult height, and developmental progression rate rather than bone age or predicted adult height alone.

There are differences in the accuracy and applicability of the methods for predicting adult height among people with different health states. Ostojic [31] reported that the difference between the predicted adult height using the TW3 method and final adult height in young male athletes was not significant; the correlation between predicted adult height and final adult height was 0.96 [31]. Rohani et al. [17] conducted a study of 15 boys with constitutional delay in growth and puberty and reported that the BP method overestimated the adult height of the boys by 5 cm. In a French study, the BP method overestimated the adult height of girls with untreated idiopathic central precocious puberty (*n*=55) by 1.4 cm and underestimated the adult height of girls with treated central precocious puberty (*n*=71) by 0.9 cm [32]. The correlation between final adult height and predicted adult height was lower in girls with treated central precocious puberty (r=0.43) than that in girls with untreated central precocious puberty (r=0.59) [32]. The results of these studies fully demonstrate the need to validate the accuracy and applicability of adult height prediction methods in the same racial and disease population.

This study complements research on the accuracy of the BP, TW3 and CH05 methods in girls with early puberty in southern China. In the present study, we showed that predicted adult height assessed by TW3 was close to adult height, whereas CH05 and BP underestimated the adult height by 2.9±3.6 cm and 1.3±3.8 cm, respectively. The TW3 method had the highest accuracy and lowest degree of dispersion. The predicted adult height obtained by TW3 had the highest correlation with adult height; therefore, it seems that the TW3 method is more suitable than the CH05 and BP methods for predicting adult height in girls with early puberty. However, TW3 tends to overestimate adult height when the bone age is younger (6–8 years), with an average overestimation of 3.4±2.9 cm. Pediatricians need to be aware of this when using the TW3 method in girls with early puberty and a younger bone age.

The efficiency of the AI systems in evaluating bone age makes them popular in the medical field. Compared with manual evaluation of bone age, which takes 2–8 min, AI-assisted bone age evaluation only takes 1–2 s [26, 27]. Machine learning algorithms for predicting adult height have been reported using growth measurements before the age of 6 years [33]. Considering that changes in growth during puberty cannot be predicted during childhood, considering factors such as age at pubertal onset, tempo of pubertal progression and bone age might be necessary in developing an AI system suitable for predicting adult height in girls with early puberty.

One of the strengths of this study is that the subjects were from the same disease population, and the sample size was large. Furthermore, it not only evaluated three bone age assessment methods but also assessed three adult height prediction methods by comparing predicted adult height with observed adult height. The limitations of this study include its retrospective nature and that it involved a limited number of girls who had reached adult height. Additionally, we did not propose improvements to current methods for assessing bone age and predicting adult height.

## Conclusion

The difference among the bone ages as measured by the three methods increased with increasing bone age, but the corresponding method for predicting adult height was more accurate when the bone age was older: The TW3 method might be more suitable for adult height prediction in girls with early puberty compared with the CH05 and BP methods, and adult height prediction methods are less accurate with younger bone age (6–11 years) than with older bone age, which is the time when girls seek medical attention and consider clinical decisions. Methods for predicting adult height should be optimized for populations of the same ethnicity and disease.

## References

[1] Mul D, Oostdijk W, Drop SLS (2002). Early puberty in girls. Best Pract Res Clin Endocrinol Metab.

[2] Pubertal Study Group of the Subspecialty Group of Endocrinologic, Hereditary and Metabolic Diseases, Society of Pediatrics, Chinese Medical Association (2010). Secondary sexual characteristics and menses in urban Chinese girls. Chin J Endocrinol Meta.

[3] Wang L, Su Z, Wang Q (2020). Survey of pubertal development among 6- to 16-year-old children in Shenzhen. Chin J Child Health Care.

[4] Eckert-Lind C, Busch AS, Petersen JH (2020). Worldwide secular trends in age at pubertal onset assessed by breast development among girls: a systematic review and meta-analysis. JAMA Pediatr.

[5] O’Sullivan E, O’Sullivan M (2002). Precocious puberty: a parent’s perspective. Arch Dis Child.

[6] Su PH, Huang JY, Li CS, Chang HP (2020). The age distribution among children seeking medical treatment for precocious puberty in Taiwan. Int J Environ Res Public Health.

[7] Chen Y, Liu J (2021). Do most 7- to 8-year-old girls with early puberty require extensive investigation and treatment?. J Pediatr Adolesc Gynecol.

[8] Lazar L, Phillip M (2012). Pubertal disorders and bone maturation. Endocrinol Metab Clin N Am.

[9] Tanner JM, Healy MJR, Goldstein H, Cameron N (2001). Assessment of skeletal maturity and prediction of adult height — TW3 method.

[10] Greulich WW, Pyle SI (1959). Radiographic atlas of skeletal development of the hand and wrist.

[11] Creo AL, Schwenk WF (2017). Bone age: a handy tool for pediatric providers. Pediatrics.

[12] Shaoyan Z (2015). The standards of skeletal age in hand and wrist for Chinese-China 05 and its applications.

[13] Zhang SY, Liu LJ, Wu ZL (2008). Standards of TW3 skeletal maturity for Chinese children. Ann Hum Biol.

[14] Bayley N, Pinneau SR (1952). Tables for predicting adult height from skeletal age: revised for use with the Greulich-Pyle hand standards. J Pediatr.

[15] Hsieh CW, Liu TC, Jong TL, Tiu CM (2013). Long-term secular trend of skeletal maturation of Taiwanese children between agricultural (1960s) and contemporary (after 2000s) generations using the Tanner-Whitehouse 3 (TW3) method. J Pediatr Endocrinol Metab.

[16] Perkins JM, Subramanian SV, Davey Smith G, Özaltin E (2016). Adult height, nutrition, and population health. Nutr Rev.

[17] Rohani F, Alai MR, Moradi S, Amirkashani D (2018). Evaluation of near final height in boys with constitutional delay in growth and puberty. Endocr Connect.

[18] Oron T, Lebenthal Y, de Vries L (2012). Interrelationship of extent of precocious adrenarche in appropriate for gestational age girls with clinical outcome. J Pediatr.

[19] Erbaş İM, Ölmez Z, Paketçi A (2021). Comparison of the effectiveness of adult height prediction methods in children with growth hormone deficiency. Endocr Res.

[20] Reinehr T, Carlsson M, Chrysis D, Camacho-Hübner C (2020). Adult height prediction by bone age determination in children with isolated growth hormone deficiency. Endocr Connect.

[21] German A, Shmoish M, Belsky J, Hochberg Z (2018). Outcomes of pubertal development in girls as a function of pubertal onset age. Eur J Endocrinol.

[22] Prokop-Piotrkowska M, Marszałek-Dziuba K, Moszczyńska E (2021). Traditional and new methods of bone age assessment — an overview. J Clin Res Pediatr Endocrinol.

[23] Booz C, Yel I, Wichmann JL (2020). Artificial intelligence in bone age assessment: accuracy and efficiency of a novel fully automated algorithm compared to the Greulich-Pyle method. Eur Radiol Exp.

[24] Thodberg HH, Thodberg B, Ahlkvist J, Offiah AC (2022). Autonomous artificial intelligence in pediatric radiology: the use and perception of BoneXpert for bone age assessment. Pediatr Radiol.

[25] Bowden JJ, Bowden SA, Ruess L (2022). Validation of automated bone age analysis from hand radiographs in a north American pediatric population. Pediatr Radiol.

[26] Wang F, Gu X, Chen S (2020). Artificial intelligence system can achieve comparable results to experts for bone age assessment of Chinese children with abnormal growth and development. PeerJ.

[27] Zhou XL, Wang EG, Lin Q (2020). Diagnostic performance of convolutional neural network-based Tanner-Whitehouse 3 bone age assessment system. Quant Imaging Med Surg.

[28] Wang YM, Tsai TH, Hsu JS (2020). Automatic assessment of bone age in Taiwanese children: a comparison of the Greulich and Pyle method and the Tanner and Whitehouse 3 method. Kaohsiung J Med Sci.

[29] Yao L (2018). Comparative study of three common digital radiograph bone age evaluation criteria. J Beijing Sport Univ.

[30] Zhang A, Sayre JW, Vachon L (2009). Racial differences in growth patterns of children assessed on the basis of bone age. Radiology.

[31] Ostojic SM (2013). Prediction of adult height by Tanner-Whitehouse method in young Caucasian male athletes. QJM.

[32] Giabicani E, Lemaire P, Brauner R (2015). Models for predicting the adult height and age at first menstruation of girls with idiopathic central precocious puberty. PLoS One.

[33] Shmoish M, German A, Devir N (2021). Prediction of adult height by machine learning technique. J Clin Endocrinol Metab.

